# Developing diagnostic assessment of breast lumpectomy tissues using radiomic and optical signatures

**DOI:** 10.1038/s41598-021-01414-z

**Published:** 2021-11-08

**Authors:** Samuel S. Streeter, Brady Hunt, Rebecca A. Zuurbier, Wendy A. Wells, Keith D. Paulsen, Brian W. Pogue

**Affiliations:** 1 Thayer School of Engineering, Dartmouth College, 14 Engineering Dr., Hanover, NH 03755 USA; 2 Department of Radiology, Dartmouth-Hitchcock Medical Center, 1 Medical Center Dr., Lebanon, NH 03756 USA; 3 Department of Pathology and Laboratory Medicine, Dartmouth-Hitchcock Medical Center, 1 Medical Center Dr., Lebanon, NH 03756 USA; 4 Norris Cotton Cancer Center, Dartmouth-Hitchcock Medical Center, 1 Medical Center Dr., Lebanon, NH 03756 USA

**Keywords:** Breast cancer, Translational research, Biomedical engineering, Imaging and sensing

## Abstract

High positive margin rates in oncologic breast-conserving surgery are a pressing clinical problem. Volumetric X-ray scanning is emerging as a powerful ex vivo specimen imaging technique for analyzing resection margins, but X-rays lack contrast between non-malignant and malignant fibrous tissues. In this study, combined micro-CT and wide-field optical image radiomics were developed to classify malignancy of breast cancer tissues, demonstrating that X-ray/optical radiomics improve malignancy classification. Ninety-two standardized features were extracted from co-registered micro-CT and optical spatial frequency domain imaging samples extracted from 54 breast tumors exhibiting seven tissue subtypes confirmed by microscopic histological analysis. Multimodal feature sets improved classification performance versus micro-CT alone when adipose samples were included (AUC = 0.88 vs. 0.90; *p*-value = 3.65e−11) and excluded, focusing the classification task on exclusively non-malignant fibrous versus malignant tissues (AUC = 0.78 vs. 0.85; *p*-value = 9.33e−14). Extending the radiomics approach to high-dimensional optical data—termed “optomics” in this study—offers a promising optical image analysis technique for cancer detection. Radiomic feature data and classification source code are publicly available.

## Introduction

Breast cancer is the second-leading cause of cancer death among women in the United States^1^. At the time of diagnosis, the disease is most frequently early-stage, localized cancer, which is predominately treated with surgical resection followed by radiation therapy. The goal of the surgical procedure, termed a lumpectomy or breast-conserving surgery (BCS), is to remove the malignancy with a surrounding layer or margin of non-cancerous tissue. An ideal BCS procedure results in cancer-free tissue on the margins (i.e., negative margins), an outcome known to provide the best prognosis^2–5^. Determining the final margin status of a BCS resection takes a day or more to complete. Depending on clinical context, one or more positive margins confirmed by histopathology may necessitate a re-excision procedure to remove residual disease, given the known association of positive margins with ipsilateral breast tumor recurrence^2,6–9^. Today, ~ 20% of BCS procedures require a follow-up re-excision due to positive margins^10–13^. Several studies report the negative health and financial impacts of BCS re-excision procedures to the patient and healthcare system^14–18^. For these reasons, improving intraoperative margin assessment in BCS is a pressing clinical need. In this study, volumetric imaging by X-rays and surface imaging by optical light were combined in a classification pipeline following conventions described in radiomics. The purpose of the study was to demonstrate how this approach might improve diagnostic assessment of malignancy in breast lumpectomy tissues, and in so doing, provide the foundational work needed to extend the approach to margin assessment in the future.

Standard of care margin assessment techniques include gross tissue inspection by the surgeon^19,20^, projection X-ray or radiographic specimen imaging^21^, and at some medical centers, frozen section pathology^22,23^, imprint cytology^24,25^, and post-excision cavity shaving^21^. Gross tissue inspection is useful for detecting palpable invasive cancers but is not as effective at detecting primary lesions intertwined with dense fibroglandular tissue or DCIS, which frequently presents as a cluster of macrocalcifications without a localized mass^26,27^. Projection (i.e., two-dimensional) X-ray imaging coupled with intraoperative reading by a radiologist is a mainstay for analyzing margins, and X-rays offer excellent contrast between adipose and fibrous tissue and sensitivity to microcalcifications^28^. Furthermore, recent advances in volumetric X-ray imaging (i.e., computed tomography and tomosynthesis) have demonstrated sensing of all six anatomical margins of a specimen in a clinically relevant timeframe with high spatial resolution^29–31^. For this reason, volumetric X-ray imaging is emerging as a powerful ex vivo specimen scanning technology. However, a key limitation of X-ray imaging is its inability to differentiate normal, abnormal benign, and malignant fibrous tissues that may be relevant to diagnosis, thereby posing the risk of frequent false positives^28,29^. Intraoperative frozen section pathology and imprint cytology reduce positive margin rates, but the approaches are not widely adopted, because they are resource-intensive (i.e., requiring pathology staff in the operating room or surgical suite during the procedure) and suffer from slow turnaround times^23,32,33^. Post-excision cavity shaving is also effective at reducing positive margin rates, requiring ≤ 10 min in the operating room with minimal impact to patient cosmesis^34,35^. However, positive margin rates after cavity shaving are variable (6–24%)^34,36–41^, suggesting alternative or complementary margin assessment techniques are still needed.

Volumetric X-ray imaging coupled with a second imaging modality could be an attractive solution for rapidly analyzing an entire BCS specimen with increased contrast to fibrous tissues on the margin^32,42^. Pradipta et al*.* reviewed an array of margin assessment techniques^32^, ranging from intraoperative ultrasonography, bioimpedance spectroscopy, and ex vivo magnetic resonance imaging to a host of optical techniques, including optical coherence tomography, ultraviolet-photoacoustic microscopy, and fluorescence probes. Each technique offers advantages and disadvantages. DiCorpo et al*.* reported that the average surface area of a BCS specimen is ~ 45 cm^2^ ^30^. A limitation of many proposed techniques is their relatively small field of view and/or long scan time, such that analyzing the entire surface of a specimen would be too time-consuming for clinical translation. Other limitations include: the need for exogenous contrast agents that must preferentially accumulate in target tissues and be proven safe for human use; or lack of evidence demonstrating efficacy when analyzing realistic BCS specimens, which can be amorphous, variable in size, and heterogeneous with a combination of adipose, fibroglandular, and potentially malignant tissues on the margin^42^.

In this study, breast tumor tissues were imaged with spatially co-registered micro-computed tomography (micro-CT) scanning and multi-wavelength spatial frequency domain imaging (SFDI). SFDI is a wide-field, noncontact, and rapid optical imaging modality first introduced by Cuccia et al*.*^43^. The technique involves the projection of one-dimensional, sinusoidal patterns of light (each at a discrete spatial frequency) onto the tissue surface and acquisition of the reflected light. At each spatial frequency, three phase-shifted patterns are projected and imaged. Each set of three phase-shifted images creates a demodulated reflectance map corresponding to the wavelength(s) and spatial frequency of illumination. Additional background information related to the optical imaging modality is available (Supplementary Material Appendix 1), and the interested reader is directed to a recent review of the modality^44^.

Previous studies have coupled machine learning models with SFDI data to classify or predict different breast tissues. One study used SFDI-derived optical scattering properties of breast tissue and an explicit mathematical model to predict the epithelial, stromal, and adipose fractions of breast tumor samples^45^. Another study applied high spatial frequency, monochromatic SFDI and a limited set of textural features to classify pairs of breast tumor subtypes using a support vector machine classifier with accuracies ranging from 55 to 95%^46^. Deep learning methods have also determined optical properties from raw SFDI data^47–49^. The use of a limited set of image features may not probe the image data sufficiently to extract the most useful signatures for differentiating tissues. On the other hand, the complexity of deep learning methods hinders model interpretation and thus limits potential for clinical translation. To address these issues, this study used a supervised machine learning pipeline based on a large number of Image Biomarker Standardization Initiative (IBSI)-compliant radiomic features^50^. The pipeline tested the ability of micro-CT alone, SFDI alone, and the combination of micro-CT and SFDI data to classify malignant and non-malignant image samples extracted from wide field-of-view images of breast tumors.

Radiomics involves the quantification of many image features, mining the features to determine diagnostic signatures not readily discerned by visual inspection, and subsequently using the features to build classification models to inform clinical decision making^51,52^. The “radiomics approach” is frequently applied to conventional medical imaging modalities (e.g., CT, magnetic resonance imaging, positron emission tomography). Applying it to optical imaging data is termed “optomics” here, extending the “omic” concept to image features extracted from wide field-of-view optical images. The goals of this study were to: first, determine whether combining X-ray micro-CT and optical SFDI image data improves malignancy classification relative to micro-CT alone based on an “omics” approach; and second, identify the most useful radiomic and optomic features for classifying malignant breast tissues.

Portions of the data collected through this imaging protocol have been analyzed in previous studies^46,49,53,54^. Specifically, SFDI data have demonstrated statistical differences between normal, abnormal benign, and malignant tissue subtypes based on color analysis and diffuse optical properties^53^, and separately, using texture analysis of monochromatic (i.e., 490 nm), high spatial frequency (1.37 mm^−1^) data^46^. SFDI data were also used in a deep learning framework, through which optical properties were approximated directly from raw SFDI data^49^. Monochromatic, high frequency SFDI and micro-CT data were also used in a comparative study to quantify differences in the wide field-of-view optical and micro-CT images^54^. The study found that optical imaging revealed intra-tumoral morphology and malignant-fibrous tissue boundaries that were occult to micro-CT scanning. The present work extends these prior contributions significantly with two key advances: first, by incorporating multi-wavelength, multi-spatial frequency SFDI *and* micro-CT data into the analysis; and second, by evaluating the image data with a supervised machine learning pipeline to quantify binary malignancy classification performance.

## Results

### Classification using radiomics and optomics

Adipose was the most frequent tissue type in the breast tumor dataset (Supplementary Material Table S1). Relative to fibroglandular and epithelial components, adipose tissue is straightforward to identify by gross surgical inspection and is readily evident with micro-CT scanning^29,54^. To test the hypothesis that including adipose samples improves performance, the classification pipeline focused on two scenarios: one that included adipose tissue samples and one that excluded them.

**Figure 1:**
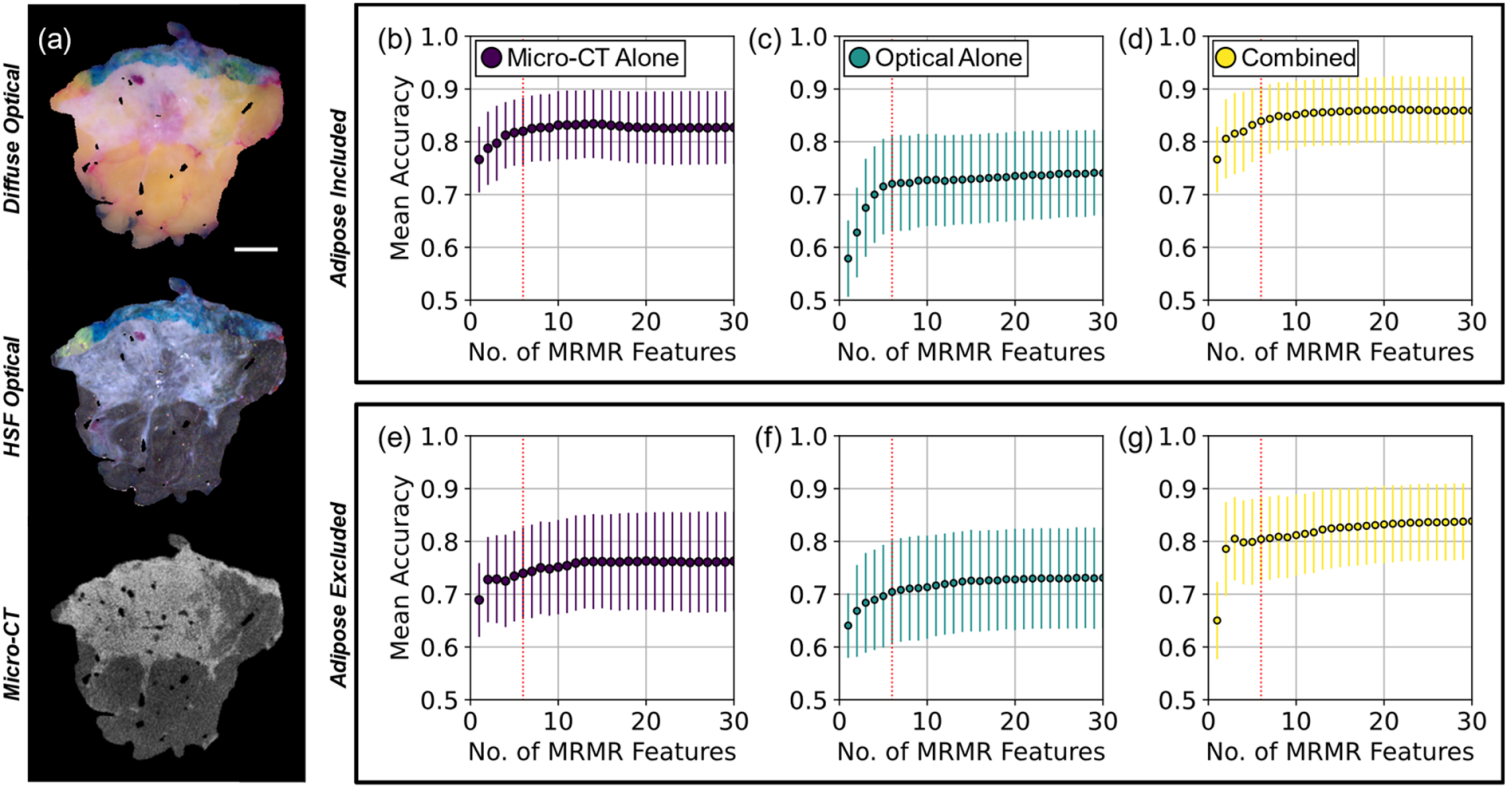
Mean classification accuracy derived from 5 × 5 mm sub-image samples as a function of the number of optimal MRMR features used in *n* = 1000 Monte Caro cross-validation splits. (**a**) Diffuse optical reflectance, high spatial frequency (HSF, 1.37 mm^−1^) optical reflectance, and a micro-CT slice of a representative tissue specimen with 1-cm scalebar. Mean accuracies when adipose tissue was included (top row, **b**–**d**) and excluded (bottom row, **e**–**g**). Dashed red vertical lines mark six features, the threshold at which the percent change in mean accuracy dropped below 1% for combined classification in (**d**) and (**g**). Error bars represent ± one standard deviation.

Figure 1a shows wide field-of-view optical and micro-CT imagery of a representative tissue specimen. Non-malignant versus malignant classification accuracy is shown for cases when adipose tissue was included (Fig. 1b–d) and excluded (Fig. 1e–g). Accuracies are plotted with respect to the number of optimal features selected by minimum redundancy, maximum relevance (MRMR)^55^. Sub-image samples of tissue ranged in size from 2 × 2 to 5 × 5 mm and were extracted from wide field-of-view regions of interest (ROIs) defining distinct tissue subtypes in each specimen. Only results from 5 × 5 mm sub-image samples are presented here, because this sub-image size provided the best classification performance overall (Supplementary Material Appendix 2 and Fig. S1). A 1% change in average accuracy determined an appropriate minimum number of features to use in each case. Based on the combined radiomic/optomic classification accuracy (Fig. 1d,g), the 1% change in average accuracy threshold required six features, when adipose tissue was both included and excluded. Therefore, subsequent analysis focused on results derived from 5 × 5 mm sub-image samples and with six radiomic/optomic features. Supplementary Materials Table S2 reports classification performance (i.e., accuracy, recall, precision, receiver operating characteristic (ROC) area under the curve (AUC)) for 5 × 5 mm sub-image samples and six total features. When adipose tissue was included and using six features, mean accuracies achieved by micro-CT features alone, optical features alone, and combined micro-CT and optical features were 82% (Fig. 1b), 72% (Fig. 1c), and 84% (Fig. 1d), respectively. When adipose tissue was excluded, mean accuracies decreased to 74% (Fig. 1e), 70% (Fig. 1f), and 80% (Fig. 1g) for the same respective feature sets.

**Figure 2:**
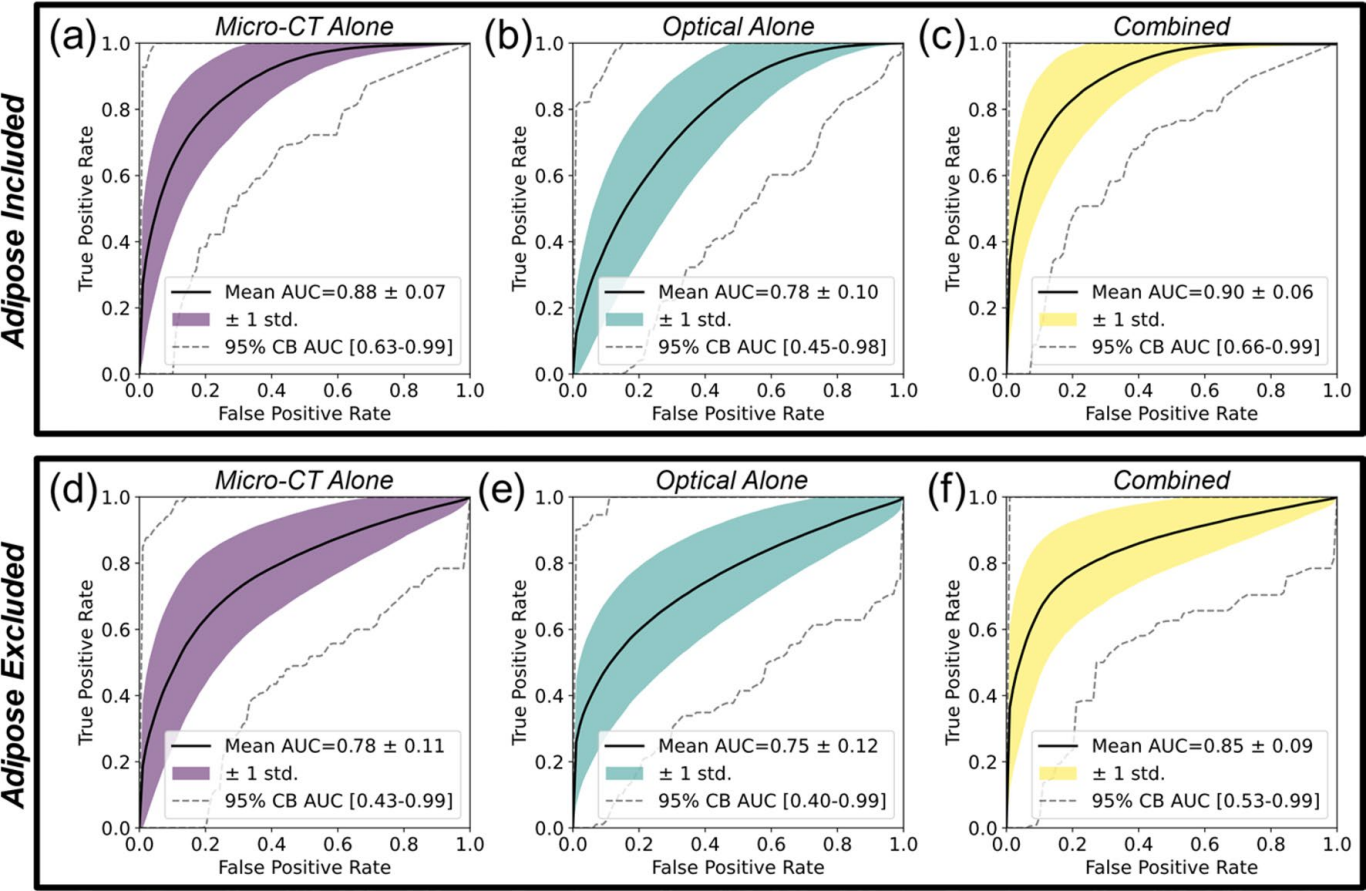
ROC curve analysis based on six radiomic/optomic features derived from 5 × 5 mm sub-image samples. Adipose tissue was included (top row) and excluded (bottom row). Each shaded region depicts two-dimensional one standard deviation from the mean ROC curve (solid black). AUC 95% confidence bands (CBs) contain 95% of the *n* = 1000 ROC curves that fell closest to the mean curve in each subplot (dashed black).

Figure 2 compares average ROC curves of optimized classifiers for micro-CT alone, optical alone, and combined feature sets. Classification performance decreased when adipose tissue samples were excluded (compare Fig. 2a with d, Fig. 2b with e, and Fig. 2c with f). Micro-CT data alone performed better than optical data alone when adipose samples were included (AUC = 0.88 vs. 0.78, *p* = 3.30e−13), but the two performed similarly when adipose samples were excluded (AUC = 0.78 vs. 0.75, *p* = 0.42). Classification performance using both modalities was better than using either modality alone (e.g., when adipose tissue was included: micro-CT vs. combined, AUC = 0.88 vs. 0.90, *p* = 3.65e−11; optical vs. combined, AUC = 0.75 vs. 0.90, *p* = 3.60e−36). Improvement in combined feature performance relative to micro-CT alone was more pronounced when adipose samples were excluded (micro-CT vs. combined, AUC = 0.78 vs. 0.85, *p* = 9.33e−14). ROC curves reflect relatively high variance depicted by one standard deviations (shaded regions) and 95% confidence bands (dashed lines), providing a visual depiction of how individual Monte Carlo CV splits performed.

### Selected feature importance

**Figure 3:**
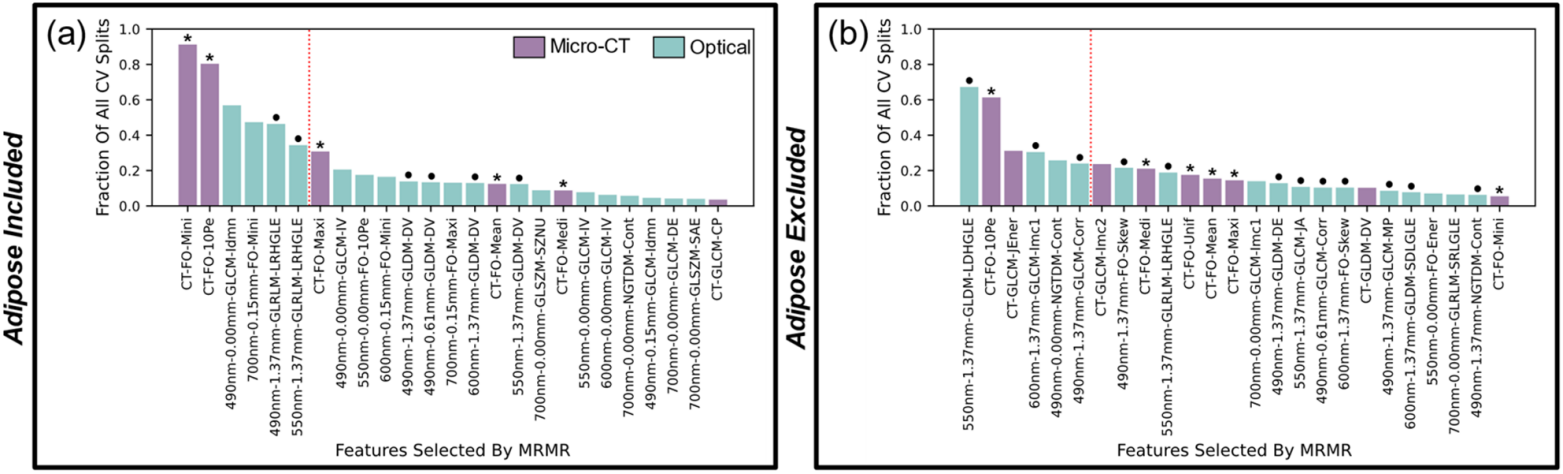
Most frequently selected features by MRMR using combined radiomic/optomic features derived from 5 × 5 mm sub-image samples when adipose tissue samples were (**a**) included and (**b**) excluded. These subplots relate directly to classification performances shown in Fig. 2c,f, respectively. Vertical axes signify the fraction of all *n* = 1000 Monte Carlo CV splits that MRMR selected each feature. Asterisks (*) signify first-order micro-CT features. Dots (•) signify high spatial frequency (0.61 or 1.37 mm^−1^) reflectance, SFDI-derived features. Twenty-five features are listed in each subplot, though additional features were selected less frequently. A complete listing of radiomic/optomic feature abbreviations can be found in Supplementary Material Appendix 5.3.

The radiomic and optomic features selected by MRMR in each of *n* = 1000 splits were tabulated. Figure 3 shows the distribution of these features for combined data classification and highlights the fact that both micro-CT and SFDI features were selected for inclusion in the optimal subset of features. This trend was true both when adipose tissue samples were included (Fig. 3a) and excluded (Fig. 3b). (These features correspond to the classification performances shown in Fig. 2c,f, respectively.) Two trends can be inferred from Fig. 3. First, the most important micro-CT radiomic features were first-order histogram statistics (noted by asterisks in the figure), which contain intensity information alone (i.e., no spatial information). This trend was true independent of whether adipose tissue was included. Second, important SFDI optomic features were generally derived from high spatial frequency reflectance (illumination frequencies of 0.61 and 1.37 mm^−1^; noted by dots in the figure), particularly when adipose tissue was omitted from the classification task. Notably, all but two of the high spatial frequency optomic features in Fig. 3 were derived from second- and higher-order pixel statistics, which depend on the spatial relationships between multiple pixels, and thus, contain textural information.

**Figure 4:**
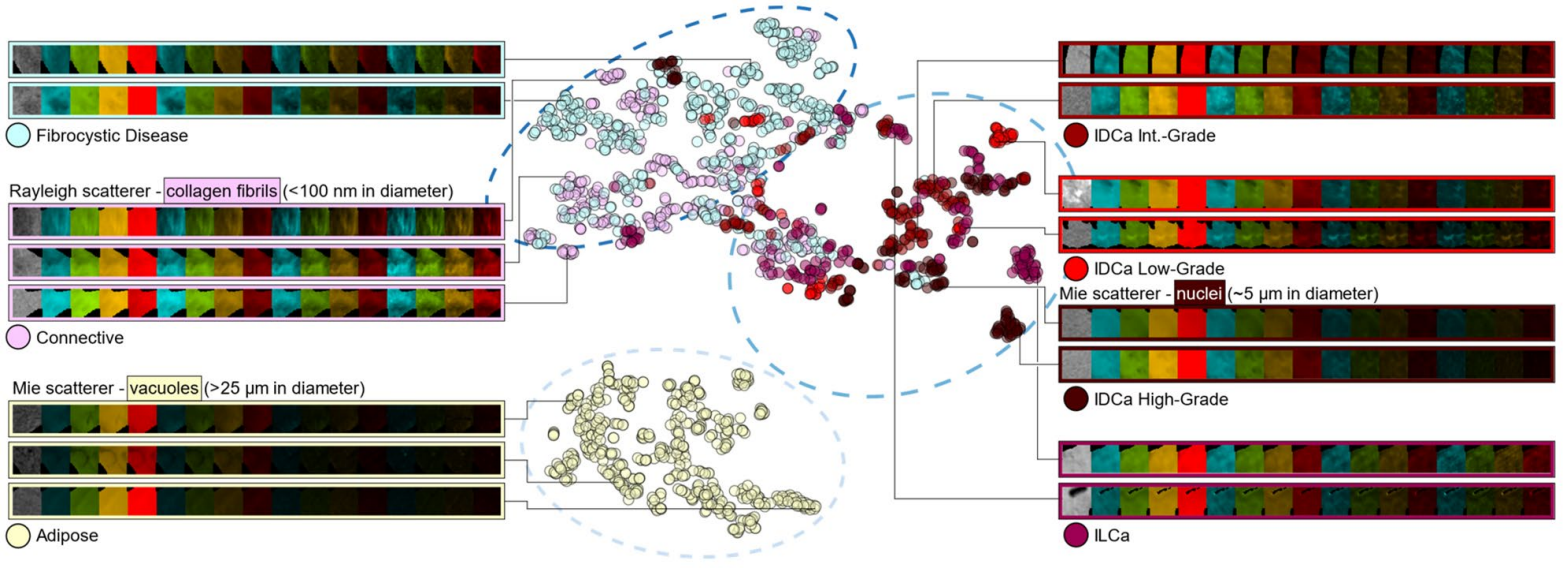
T-distributed stochastic neighbor embedding using six optimal MRMR radiomic/optomic features (i.e., top features shown in Fig. 3b). Dashed ellipses qualitatively delineate three clusters of samples, which can be related to hierarchical clusters depicted in Supplementary Material Fig. S2. Image channel labels show the micro-CT sub-image on the left (grayscale), followed by calibrated reflectance optical channels with spatial frequency increasing from left to right (0.00, 0.15, 0.61, then 1.37 mm^−1^). Coloring of optical channels illustrates the wavelength of light, increasing from left to right (490, 550, 600, then 700 nm) and repeated for each spatial frequency. IDCa = invasive ductal carcinoma. ILCa = invasive lobular carcinoma.

A t-distributed stochastic neighbor embedding plot, shown in Fig. 4, reduces the dataset to a two-dimensional embedding of only six optimal radiomic/optomic features (using the six most frequently selected features shown in Fig. 3b). Each sub-image sample is color-coded by tissue subtype, and representative samples are labeled with the associated micro-CT and optical image channels. Figure 4 suggests separability of normal (i.e., connective tissue) and abnormal benign (i.e., fibrocystic disease) samples from malignant tissue subtypes is possible when only six radiomic/optomic features are used. Adipose tissue is also clustered effectively under these conditions.

High-dimensional radiomic datasets are frequently visualized using hierarchically clustered heatmaps^51,52,56^, and such a visualization is available for the dataset in this study (Supplementary Material Appendix 3 and Fig. S2). Notably, the global structure shown in Fig. S4 is maintained by Fig. 4 using six instead of 1564 radiomic/optomic features. The image channel labels in Fig. 4 exemplify representative tissue subtype image signatures: first, adipose tissue yields relatively low linear attenuation coefficient values in the micro-CT sub-image relative to all other subtypes^54^; second, adipose tissue and high-grade invasive ductal carcinoma are predominantly characterized by Mie scattering vacuoles (> 25 μm in diameter) and nuclei (~ 5 μm in diameter), respectively, explaining the relatively low high spatial frequency optical reflectance for these samples^57^; and third, connective tissue and fibrocystic disease contain collagen fibril structures (< 100 μm in diameter) that are strong Rayleigh-type scatterers, giving rise to an increased backscatter signal in these optical sub-images^57^.

## Discussion

Analyses indicate overall mean accuracy and AUC achieved were 84% and 0.90 (80% and 0.85 without adipose tissue), respectively, using only six optimal radiomic/optomic features. These results appear to be the first application of radiomics to multi-wavelength, multi-spatial SFDI data and represent a novel approach for validating and extending radiomic feature analysis to optical imaging data. They provide a proof of principle for how radiomic and optomic features can be combined to improve overall classification accuracy. Other important contributions from this study include appropriate image data normalization to linear attenuation coefficient and calibrated reflectance values and use of IBSI-compliant image features in the classification pipeline, considerations that lend to study rigor and reproducibility. Finally, use of defined image features within a supervised machine learning pipeline provides increased model interpretability relative to deep learning methods of tissue classification.

As described in the Introduction, previous studies used portions of the same breast cancer dataset used in this study. The new method revealed in this work relative to previous studies is multimodal radiomic/optomic analysis. The radiomics approach is often deployed with other imaging modalities relevant to the diagnosis and monitoring of breast cancer, mainly magnetic resonance imaging, CT, positron emission tomography, and/or ultrasound. Most studies demonstrate radiomics utility in identifying malignant lesions in the setting of pre-operative breast cancer diagnosis, and recently published classification performance AUCs range from 0.57 to 0.98 for this task^58,59^. The work described here suggests that wide field-of-view optical images of breast tumors likely contain useful image features, especially textural information, relevant to diagnosis. The optomics approach provides an alternative to optical property quantification, a process that requires tissue model assumptions and can be computationally intensive to implement (e.g., ~ 1 h for a 2.5 cm × 2.5 cm tissue sample^57^). The study also demonstrates that the combined radiomics/optomics approach is synergistic for automated diagnostic assessment of breast tissues. Additional findings revealed in this work relative to previous studies include the fact that useful optical features can be extracted from a wide wavelength range (i.e., optimal features span 490–700 nm in Fig. 3a and 490–600 nm in Fig. 3b) and the entire spatial frequency range in the dataset (i.e., optimal features span 0.00–1.37 mm^−1^ in Fig. 3a,b). Notably, the top six features selected by MRMR differed significantly depending on whether adipose tissue was included in the classification task. When adipose was included (Fig. 3a), the top two features were micro-CT first-order histogram statistics. This result is expected given the stark difference in radiodensity between adipose and all other tissues in the breast cancer specimens; purely differences in pixel intensity were sufficient to separate adipose from all other tissues. When adipose was excluded (Fig. 3b), three of the top six features were textural features derived from high spatial frequency optical reflectance, suggesting that this feature type provides value for differentiating tissues that exhibit similar radiodensities. Nevertheless, the second most important feature when adipose tissue was excluded was a micro-CT first-order statistic, reinforcing the claim that micro-CT and optical imaging provide complementary contrast regardless of the presence of adipose tissue.

Importantly, improvements in margin assessment were not directly demonstrated in this study. Rather, freshly resected breast tumor slices were imaged to capture a range of tumor pathologies, and the focus was building differential diagnostic models. It is also necessary to distinguish cancer tissue on the margin that has been thermally denatured or burned by surgical tools during resection, processes known to alter tissue optical properties^60^. However, this limitation was not addressed in this study. Only invasive cancers were analyzed due to a lack of pre-invasive DCIS specimens in the dataset. This limitation is significant, given that relative to other malignant tissue subtypes, DCIS is responsible for one of the largest shares of positive margins leading to re-excision procedures^13^. Future studies should include DCIS samples to demonstrate efficacy in classifying this important pre-invasive subtype. Finally, future work should also involve radiomic/optomic analysis of intact BCS specimens, taking advantage of the volumetric sensing of micro-CT in combination with surface-mapped optical reflectance to evaluate the performance of this approach for sensing cancer-positive tissues at the margin. For example, the radiomic feature quantification package used in this study, PyRadiomics^56^, offers a suite of standardized, IBSI-compliant 3D shape and voxel-based radiomic features that could be extracted from sub-volumes of the micro-CT scan. Connected sub-volumes that exhibit malignant/suspicious radiomic signatures that extend to the tissue margin could then be correlated with optomic signatures from the surface tissue to identify potentially involved margins.

This study introduces an optomics paradigm for analyzing high dimensional optical image data and represents a direct and quantitative assessment of the extent to which coupling micro-CT scanning with optical imaging improves classification of malignant breast tumor tissues. Volumetric X-ray imaging is complimentary to surface SFDI, which provides additional sensing of surface tissue subtypes, especially those that are fibrous and exhibit similar radiodensities. These results warrant further research into the combination of specimen X-ray imaging with wide field-of-view, noncontact optical imaging, such as SFDI, for potentially improving intraoperative margin assessment.

## Materials and methods

### Breast tumor imaging protocol

All methods were carried out in accordance with relevant guidelines and regulations. Specimen imaging was performed at the Dartmouth-Hitchcock Medical Center (DHMC) in Lebanon, New Hampshire. The imaging protocol was approved by the Committee for the Protection of Human Subjects, the Institutional Review Board at Dartmouth College, and all aspects of the study followed the approved protocol. Tissue specimens were procured from patients electing BCS at DHMC who participated in the study under informed consent. Imaging was performed post-operatively during standard of care pathological processing in the specimen grossing laboratory and did not impact tissue processing or diagnostic reporting in any way. One representative, ~ 5-mm thick slice from each BCS tumor was selected by an experienced Pathologists’ Assistant for imaging and was de-identified and referenced only by a unique accessioning number. Each imaged slice exhibited a clear cross section of the primary tumor and surrounding tissues, thereby revealing clear regions of several normal, abnormal benign, and malignant breast tissue subtypes. Each slice was firmly positioned between clear acrylic plates, creating a flat tissue surface that mitigated specular reflection and demodulation artifacts in the SFDI data. The top surface of each imaged slice underwent standard of care specimen processing, sectioning, staining with hematoxylin and eosin, and microscopic analysis by a board-certified breast pathologist (WAW). Histologic slides corresponding to the imaged tissue surface underwent whole slide, high resolution digital imaging and were then mosaicked together to confirm wide-field tissue subtype ROIs. These microscopic ROIs were co-registered to the wide field-of-view micro-CT and optical imagery.

### Imaging system

**Figure 5:**
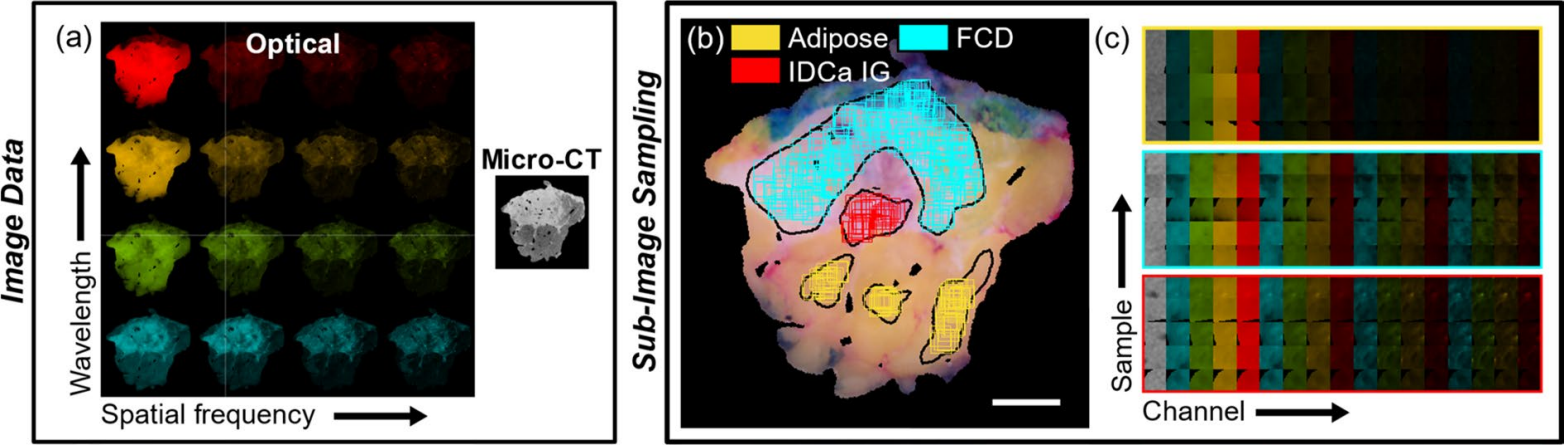
Overview of tissue imaging and sampling protocol. (**a**) Visualization of optical data collection at four wavelengths and four spatial frequencies along with micro-CT scanning of a representative tissue specimen. (**b**) Sub-image sampling from histologically confirmed regions of distinct tissue subtypes, and (**c**) sub-image samples grouped by tissue subtype (color borders) containing 17 channels of image data (16 optical, one micro-CT). FCD = fibrocystic disease. IDCa IG = invasive ductal carcinoma intermediate-grade.

Imaging was performed with a customized IVIS SpectrumCT system (PerkinElmer, Hopkinton, MA) containing a cone-beam CT in a “pancake” geometry and retrofitted optical imaging components^61^. Micro-CT scans were acquired with X-ray tube settings of 50 kVp and 1 mA with an exposure time of 100 ms/projection for a total of 720 projections. The reconstructed scan volume was 12 × 12 × 3 cm^3^ with 150 μm^3^ voxels, and the combined acquisition and reconstruction time was ~ 4 min. These settings were the same as those used in a previous micro-CT BCS specimen study^29^. The SFDI acquisition leveraged the charged coupled device camera native to the IVIS SpectrumCT system (Andor iKon, Andor Technologies Ltd, Belfast, UK) and a digital light projector (CEL5500 Fiber, Digital Light Innovations Inc., Austin, TX) retrofitted in the light-tight, fully shielded imaging cabinet for projecting the structured light patterns. The light source was a supercontinuum laser (SuperK Blue, NKT Photonics, Denmark) with a tunable line filter (SuperK Varia, NKT Photonics, Denmark). SFDI acquisition involved optical wavelengths of 490, 550, 600, and 700 nm, and illumination spatial frequencies of 0.00, 0.15, 0.61, and 1.37 mm^−1^. Thus, 16 unique wavelength-spatial frequency reflectance images were collected per specimen. SFDI acquisition and reconstruction time was ~ 8 min. Combined with the surface tissue micro-CT slice, every specimen in the dataset had 17 channels of image data (Fig. 5a).

### Image data and pre-processing

A total of 70 specimens were imaged under the approved protocol. Sixteen cases failed to meet data analysis eligibility criteria: five were excluded due to inconsistent micro-CT scan settings, six were excluded due to ambiguous histology co-registrations, one was excluded due to a small cross-sectional area (< 2 cm^2^), and four were omitted, because they presented tissue subtypes that were represented in fewer than three specimens in the dataset. Thus, 54 tumor specimens from 54 BCS patients were analyzed. From these specimens, 177 ROIs were drawn, isolating regions of seven histologically confirmed breast tissue subtypes. Of the 54 specimens, 14 contained only normal and/or abnormal benign tissues, three contained only malignant tissue, and 37 contained both normal or abnormal benign and malignant tissues. Table 1 summarizes the number of breast tumor specimens and the number of ROIs categorized by tissue subtype. The number of sub-images extracted from each ROI was proportional to the size of each ROI, providing an approximate baseline by which all ROIs were equally sampled. Figure 5b illustrates the process of sub-image sampling from the wide field-of-view tissue images. Supplementary Material Table S1 contains totals of sub-image samples categorized by subtype and by sub-image size.

**Table 1:** Breast tumor specimen and ROI totals by tissue subtype in this study.

Tissue subtype	Specimen count	ROI count	ROI Size (average ± 1 std mm^2^)
**Healthy and abnormal benign**			
Adipose tissue	49	93	43 ± 32
Normal connective tissue	13	20	85 ± 66
Fibrocystic disease	14	22	112 ± 133
**Invasive cancer**			
IDCa low-grade	10	10	46 ± 33
IDCa intermediate-grade	13	13	59 ± 56
IDCa high-grade	10	10	82 ± 58
Invasive lobular carcinoma	9	9	99 ± 102

*IDCa* invasive ductal carcinoma.

Calibrated reflectance maps were generated at each of 16 wavelength-spatial frequency settings using a previously described normalization process with a reflectance standard^61^. Micro-CT scans were converted to linear attenuation coefficient values based on a 50 kVp X-ray energy. All image data were masked to remove regions with poor tissue coupling to the top acrylic plate, thereby restricting the image analysis to flat, consistent tissue surfaces. Micro-CT scanning and SFDI were completed sequentially, without moving the tissue or acrylic plates between acquisitions, facilitating spatial co-registration between modalities. Tissue subtype ROIs were sampled over a range of square sub-image sizes (2 × 2–5 × 5 mm).

### Radiomics package and classification pipeline

The classification pipeline (summarized in Supplementary Material Fig. S3) performed binary stratification of samples as malignant or non-malignant based on radiomic and/or optomic features. Fornacon-Wood et al*.* recently reviewed several popular radiomic feature quantification software packages, identifying strengths and weaknesses of each package^62^. Here, the pipeline was implemented in the Python coding language (v3.7.9) using the PyRadiomics package (v3.0.1) for feature quantification, given that it is IBSI-compliant for reproducibility, free of cost, and open-source^56^. The pipeline involved MRMR feature selection, a random forest classifier, and Monte Carlo cross-validation with *n* = 1000 splits partitioned on the patient-level. Additional details related to the classification pipeline are available (Supplementary Material Appendix 4 and Fig. S3).

Features were quantified from only micro-CT data (1 channel), only SFDI data (16 channels), and combined data (17 channels) (Fig. 5c). A total of 92 PyRadiomic features were extracted from each image channel. Thus, up to 1,564 features were quantified from each sub-image sample. Quantified features included a range of first-, second-, and higher order pixel statistics. Supplementary Material Appendix 5 contains additional information about the features quantified, including fixed bin width considerations (Appendix 5.1, Fig. S4) and a complete list of features and associated abbreviations (Appendix 5.2).

### Statistical analysis

For every combination of pipeline parameters, ROC curves (*n* = 1000) were generated through Monte Carlo CV. Average ROC curves were derived by interpolating true positive values to a constant range of false positives across all splits. DeLong’s test determined whether differences between pairs of average ROC AUC values were statistically significant^63,64^. A *p*-value ≤ 0.05 was considered significant.

## Supplementary Information

Supplementary Information.

## Data Availability

Image data in the form of comma-separated value files, classification pipeline source code, and a PyRadiomics parameter file documenting all feature quantification settings are available with this publication in an open-source repository (https://github.com/optmed/radiomics-optomics).
