# Supplementary material for: Developing diagnostic assessment of breast lumpectomy tissues using radiomic and optical signatures
Source: Sci Rep. 2021 Nov 8;11:21832. doi: 10.1038/s41598-021-01414-z (PMC8575781; doi:10.1038/s41598-021-01414-z)
Supplement: Supplementary file 1 — Supplementary Information. [file 41598_2021_1414_MOESM1_ESM.docx]

**Supplementary Material for**

**Developing diagnostic assessment of breast lumpectomy tissues using radiomic and optical signatures**

Samuel S. Streeter*^1^, Brady Hunt^1^, Rebecca A. Zuurbier^2,4^, Wendy A. Wells^3,4^, Keith D. Paulsen^1,4^, and Brian W. Pogue^1,4^

^1^ Thayer School of Engineering, Dartmouth College, 14 Engineering Dr., Hanover, NH 03755

^2^ Dept. of Radiology, Dartmouth-Hitchcock Medical Center, 1 Medical Center Dr., Lebanon, NH 03756

^3^ Dept. of Pathology and Laboratory Medicine, Dartmouth-Hitchcock Medical Center, 1 Medical Center Dr., Lebanon, NH 03756

^4^ Norris Cotton Cancer Center, Dartmouth-Hitchcock Medical Center, 1 Medical Center Dr., Lebanon, NH 03756

*Corresponding author: [Samuel.S.Streeter.TH@Dartmouth.edu](mailto:Samuel.S.Streeter.TH@Dartmouth.edu).

**Appendix 1: Spatial frequency domain imaging**

Spatial frequency domain imaging (SFDI) is a wide-field, noncontact, structured light imaging modality first introduced by Cuccia et al.^1^ and further developed by the same group into a quantitative imaging modality based on theoretical modeling of diffuse light transport^2^. By imaging the reflectance from a range of one-dimensional sinusoidal (i.e., discrete spatial frequency) illumination patterns, the tissue’s modulation transfer function (MTF) can be measured discretely. The most common application of SFDI is optical property quantification (e.g., absorption and reduced scattering coefficients)^2,3^. Using nonlinear least squares fitting of the measured MTF to theoretical models of light transport, tissue optical properties can be measured pixel-by-pixel. Recent studies have demonstrated the potential of SFDI to differentiate breast tissues using optical properties^4,5^, particularly at high spatial frequencies of illumination, which yield surface-weighted reflectance dominated by scatter-based contrast^6^.

# Appendix 2: Classification accuracy vs. sub-image sampling size

**Fig. S1** summarizes mean classification performance with respect to three variables: number of optimal features selected by minimum redundancy, maximum relevance (MRMR) (horizontal axis), modality (datapoint color), and sub-image sample size (datapoint shape). In general, the larger sub-image samples provided the highest mean accuracy, and therefore, results in the main manuscript focused on results derived from 5 x 5 mm samples. A 1% change in mean classification performance defined an appropriate minimum number of radiomic/optomic features to include in the classification task. For classification based on combined features derived from 5 x 5 mm sub-image samples (yellow diamonds in **Fig. S1**), only six features were required both when adipose tissue was included and excluded to reach the 1% accuracy improvement threshold (dashed, vertical red lines). Note that standard deviations are omitted from **Fig. S1** for visual clarity.

**Appendix 3: Hierarchical clustering heatmap**

**Fig. S2** shows a two-dimensional hierarchically clustered heatmap of the breast tumor 5 x 5 mm sub-image samples in similar fashion to other radiomics visualizations^7–9^. Cluster 1 is characterized primarily by an adipose radiomic/optomic signature. It contains all adipose tissue samples in the dataset, and only 15% of the samples are malignant. Cluster 2 is most closely designated as a malignant signature; 43% of samples in this cluster represent various subtypes of malignancy. Cluster 3 is mostly aligned with non-malignant fibrous tissue; only 22% of its samples are malignant, while the remaining 78% are normal connective or benign fibrocystic disease samples. The “Patient” column on the left side of the figure color-codes tumor samples on the patient-level and indicates that individual patient data were dispersed between clusters. Conventional radiomics does not typically relate hierarchical clustering to subsequent classification results. **Fig. S2** establishes this connection visually; the probability of malignancy associated with each sample is plotted vertically on the left side of the heatmap, derived from all *n* = 1000 CV splits (i.e., using combined micro-CT and SFDI data, 5 x 5 mm sub-image samples, and including adipose tissue samples; the data used to create results in **Fig. 2c** and **Fig. 3a** in the main manuscript).

**Appendix 4: Classification pipeline**

The classification pipeline (summarized in **Fig. S3**) used Scikit-learn (abbreviated “sklearn,” v0.24.1), an open-source Python library for machine learning^10^. Because the distribution of tissue subtypes was variable across specimens, and significant patient/specimen-level bias existed in the dataset, an appropriate cross validation (CV) scheme was incorporated. Monte Carlo CV (using the “GroupShuffleSplit” sklearn function) was performed (*n* = 1000 times), randomly partitioning specimens on a patient-level (equivalent to specimen-level partitioning for this dataset)^11^. In each split, 20% of patients were withheld from training. Patient-level bias was mitigated by reporting performance metrics averaged over all splits. The random seed for selecting the training and testing set indices was user-defined (by setting the “random_state” argument in “GroupShuffleSplit” sklearn function), such that comparisons between different sets of parameter options were based on an identical set of 1000 CV splits. Given that the dataset contained far more non-malignant than malignant samples (**Supplementary Material Table S1**), the non-malignant training samples were randomly down-sampled to balance the training set in every split (using the “RandomUnderSampler” function from the imblearn Python package^12^, v0.8.0). Radiomic and/or optomic features corresponding to training set samples were standardized to z-scores (using the “StandardScaler” sklearn function), and the feature means and standard deviations from the training set standardized the testing set features to minimize data leakage. The number of radiomic/optomic features used in model training and testing, *k*, was varied (all values k ∈ ℤ: k ∈ [1, 30]). MRMR feature selection identified the subset of *k* features having the most correlation with the malignant class and the least correlation between *k* features in the training set^13^ (using the “mrmr” function from the pymrmre Python package^14^, v1.0.7). A random forest classifier with default settings (“RandomForestClassifier” sklearn function with default settings) was then trained and tested on each split. The choices of MRMR feature selection and random forest classifier were based on a thorough analysis by Parmar et al. that determined these selections performed well for radiomics-based classification of CT segmentations of non-small cell lung cancer^15^. Test set performance was recorded for each split in the form of classification accuracy, recall, precision, and receiver operating characteristic area under the curve.

**Appendix 5: Feature quantification using PyRadiomics**

*5.1 Fixed bin widths for gray value discretization*

Radiomic feature quantification first requires gray value discretization of the image data. For this study, a fixed bin width of 0.005 for both calibrated reflectance (unitless) and 50 kVp linear attenuation coefficient (cm^-1^) values was used. A fixed bin width rather than a fixed bin count was used given that it provides improved reproducibility when inter-sample dynamic range is variable^16^. Tixier et al. found that bin widths that resulted in 30-130 total bins yielded good reproducibility and performance^17^. All tissue pixel values extracted from the micro-computed tomography (micro-CT) and SFDI images are shown in histogram form in **Fig. S4a** and **Fig. S4b**, respectively. From these histograms, distribution percentiles and appropriate bin width values were determined. This analysis found that a fixed bin width of 0.005 fell within the 30-130 total bin range for both modalities based on the range defined by the first and ninety-nineth percentile values for each modality.

The micro-CT tissue pixel distribution in **Fig. S4a** was slightly bi-modal, which is explained by the dominant X-ray contrast observed between fibroglandular tissue and adipose tissue in breast tumors^18^. The SFDI tissue pixel distribution in **Fig. S4b** was highly skewed to low calibrated reflectance values due to the fact that the dataset included non-zero spatial frequency reflectance, which is known to yield relatively low reflectance from tissue, especially at sub-diffuse spatial frequencies (i.e., 0.61 and 1.37 mm^-1^)^4,6^.

*5.2 List of quantified PyRadiomic features and associated feature abbreviations*

Quantified features included a range of first-, second-, and higher order pixel statistics: 18 first-order (FO) histogram statistics, 23 gray level co-occurrence matrix (GLCM) features, 16 gray level run length matrix (GLRLM) features, 16 gray level size zone matrix (GLSZM) features, 14 gray level dependence matrix (GLDM) features, and five neighboring gray tone difference matrix (NGTDM) features. Although the PyRadiomics package includes a range of features quantifying two- and three-dimensional shapes from segmented ROIs, all shape features were omitted given the consistent square sub-image sampling routine implemented in this study. Furthermore, no filter-based features (e.g., Log of Gaussian, Wavelet) were included in this study. Each feature name started with a prefix denoting the modality from which it was derived: either “CT” for micro-CT or “###nm-#.##mm” for optical, where the first number depicted the optical wavelength (490, 550, 600, or 700 nm) and the second number depicted the spatial frequency of illumination (0.00, 0.15, 0.61, and 1.37 mm^-1^).

Quantified features are listed below, categorized by feature class with feature abbreviations in parentheses:

**First-order (FO) histogram statistics:**

1. 10^th^ Percentile (10Pe)
2. 90^th^ Percentile (90Pe)
3. Energy (Ener)
4. Entropy (Entr)
5. Interquartile Range (IR)
6. Kurtosis (Kurt)
7. Maximum (Maxi)
8. Mean Absolute Deviation (MAD)
9. Mean (Mea)
10. Median (Medi)
11. Minimum (Mini)
12. Range (Rang)
13. Robust Mean Absolute Deviation (RMAD)
14. Root Mean Squared
15. Skewness (Skew)
16. Total Energy (TE)
17. Uniformity (Unif)
18. Variance (Vari)

**Gray level co-occurrence matrix (GLCM) features:**

1. Autocorrelation (Auto)
2. Cluster Prominence (CP)
3. Cluster Shade (CS)
4. Cluster Tendency (CT)
5. Contrast (Cont)
6. Correlation (Corr)
7. Difference Average (DA)
8. Difference Entropy (DE)
9. Difference Variance (DV)
10. Inverse Difference (Id)
11. Inverse Difference Moment (Idm)
12. Inverse Difference Moment Normalized (Idmn)
13. Inverse Difference Normalized (Idn)
14. Informational Measure of Correlation 1 (Imc1)
15. Informational Measure of Correlation 2 (Imc2)
16. Inverse Variance (IV)
17. Joint Average (JA)
18. Joint Energy (JEner)
19. Joint Entropy (JEntr)
20. Maximal Correlation Coefficient (MCC)
21. Maximum Probability (MP)
22. Sum Entropy (SE)
23. Sum Squares (SS)

**Gray level run length matrix (GLRLM) features:**

1. Gray Level Non-Uniformity (GLNU)
2. Gray Level Non-Uniformity Normalized (GLNUN)
3. Gray Level Variance (GLV)
4. High Gray Level Run Emphasis (HGLRE)
5. Long Run Emphasis (LRE)
6. Long Run High Gray Level Emphasis (LRHGLE)
7. Long Run Low Gray Level Emphasis (LRLGLE)
8. Low Gray Level Run Emphasis (LGLRE)
9. Run Entropy (RE)
10. Run Length Non-Uniformity (RLNU)
11. Run Length Non-Uniformity Normalized (RLNUN)
12. Run Percentage (RP)
13. Run Variance (RV)
14. Short Run Emphasis (SRE)
15. Short Run High Gray Level Emphasis (SRHGLE)
16. Short Run Low Gray Level Emphasis (SRLGLE)

**Gray level size zone matrix (GLSZM) features:**

1. Gray Level Non-Uniformity (GLNU)
2. Gray Level Non-Uniformity Normalized (GLNUN)
3. Gray Level Variance (GLV)
4. High Gray Level Zone Emphasis (HGLZE)
5. Large Area Emphasis (LAE)
6. Large Area High Gray Level Emphasis (LAHGLE)
7. Large Area Low Gray Level Emphasis (LALGLE)
8. Low Gray Level Zone Emphasis (LGLZE)
9. Size Zone Non-Uniformity (SZNU)
10. Size Zone Non-Uniformity Normalized (SZNUN)
11. Small Area Emphasis (SAE)
12. Small Area High Gray Level Emphasis (SAHGLE)
13. Small Area Low Gray Level Emphasis (SALGLE)
14. Zone Entropy (ZE)
15. Zone Percentage (ZP)
16. Zone Variance (ZV)

**Gray level dependence matrix (GLDM) features:**

1. Dependence Entropy (DE)
2. Dependence Non-Uniformity (DNU)
3. Dependence Non-Uniformity Normalized (DNUN)
4. Dependence Variance (DV)
5. Gray Level Non-Uniformity (GLNU)
6. Gray Level Variance (GLV)
7. High Gray Level Emphasis (HGLE)
8. Large Dependence Emphasis (LDE)
9. Large Dependence High Gray Level Emphasis (LDHGLE)
10. Large Dependence Low Gray Level Emphasis (LDLGLE)
11. Low Gray Level Emphasis (LGLE)
12. Small Dependence Emphasis (SDE)
13. Small Dependence High Gray Level Emphasis (SDHGLE)
14. Small Dependence Low Gray Level Emphasis (SDLGLE)

**Neighboring gray tone difference matrix (NGTDM) features:**

1. Busyness (Busy)
2. Coarseness (Coar)
3. Complexity (Comp)
4. Contrast (Cont)
5. Strength (Stre)

**Supplementary Material Figures**

**
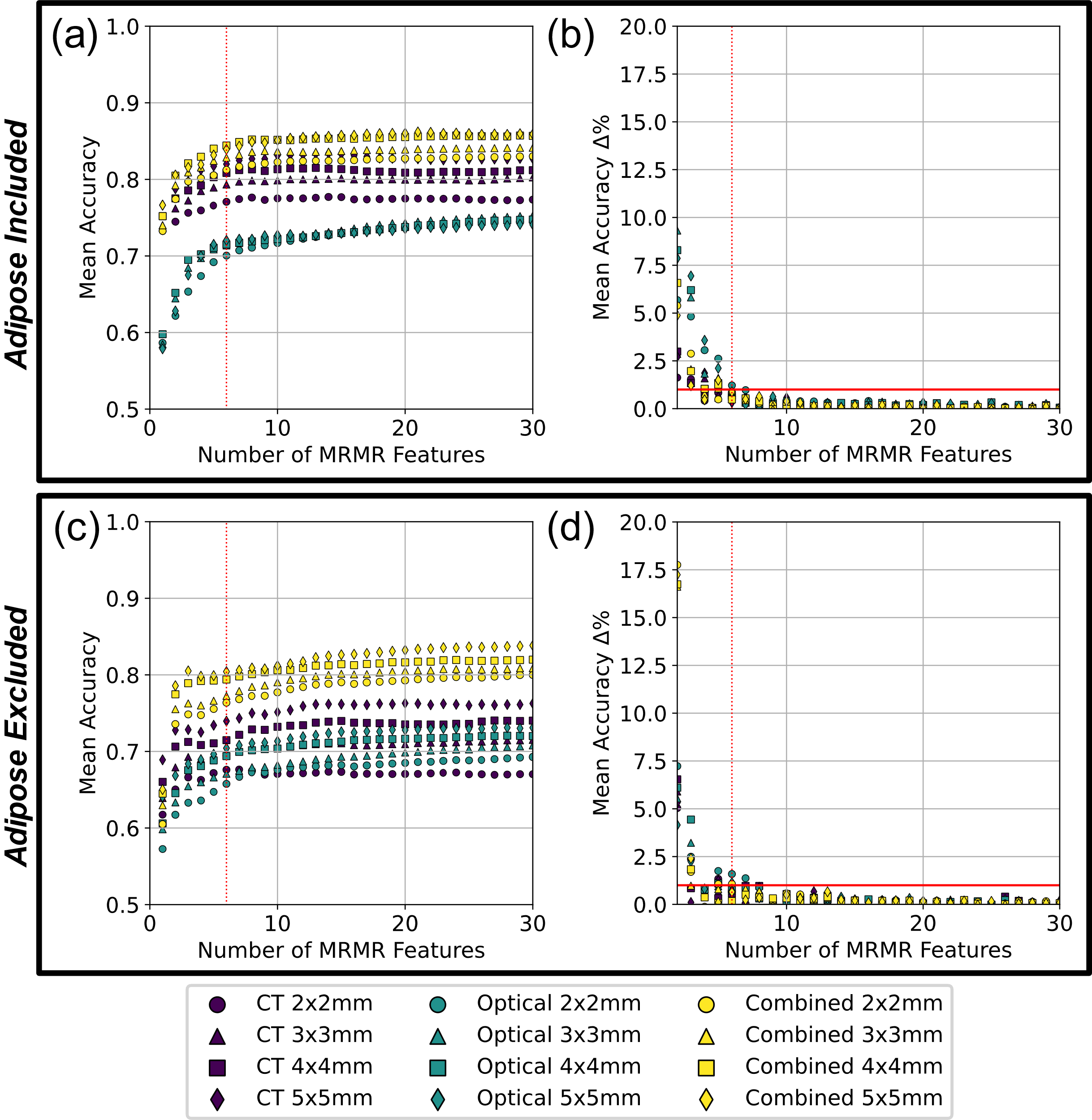
**

**Fig. S1** (a) and (c) Average binary classification accuracy with respect to the number of radiomic and/or optomic features used in each of *n* = 1000 Monte Caro cross-validation splits. Adipose tissue was included in top row and omitted in bottom row. (b) and (d) Percent change in mean accuracy versus the number of optimal features used. A threshold of 1% change in accuracy (solid, horizontal red lines) determined a suitable minimum number of optimal features necessary for the classification task (six features, dashed, vertical red lines). MRMR = Minimum redundancy, maximum relevance feature selection.


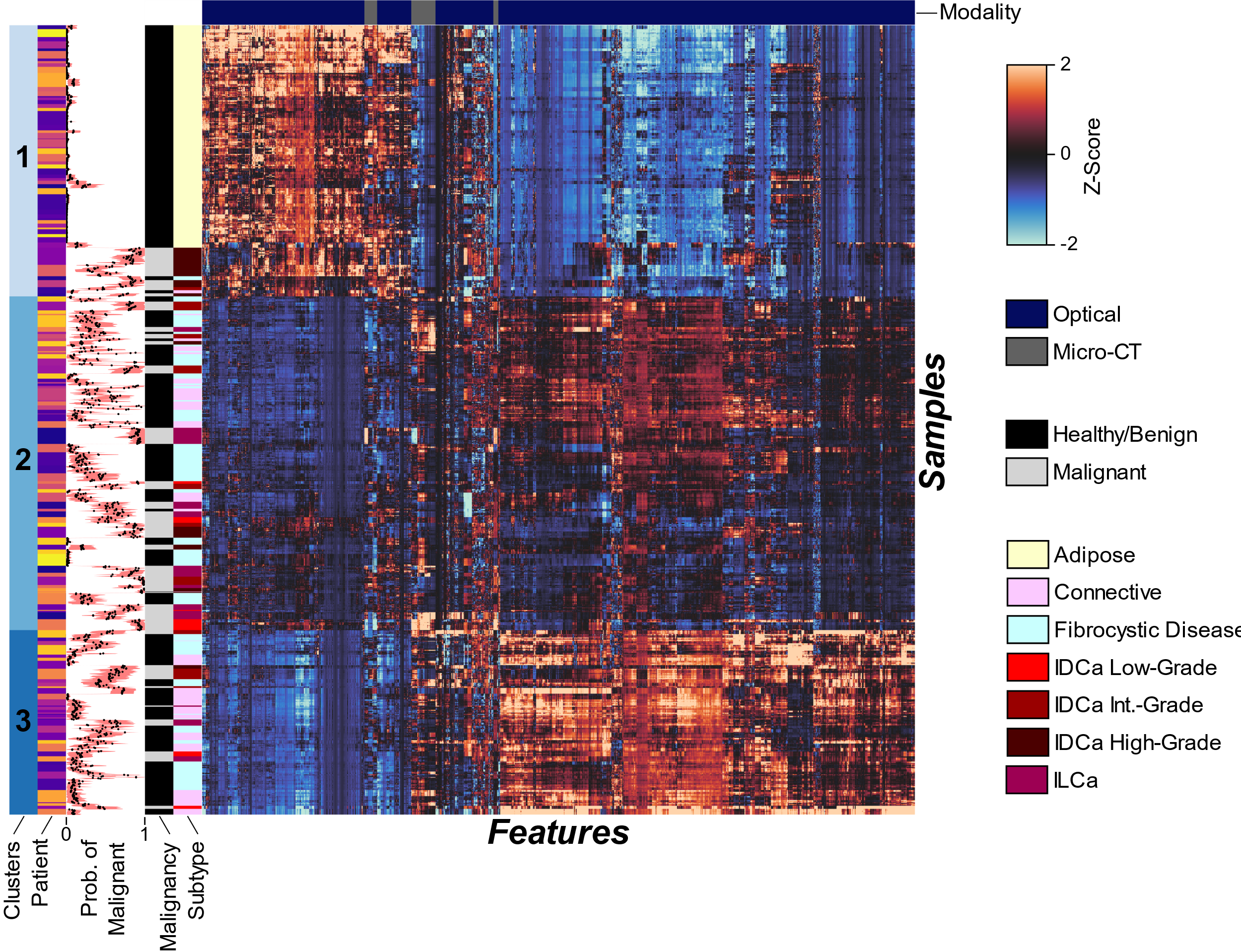


**Fig. S2** Hierarchical clustering with respect to samples (1,802 total; vertical axis) and features (1,564 total; horizontal axis) reveals natural groupings of breast tumor samples. Probability of malignancy is shown along the left side (average ± one standard deviation depicted by black dots and red shading, respectively, from all *n* = 1000 Monte Carlo CV splits). IDCa = invasive ductal carcinoma. ILCa = invasive lobular carcinoma.


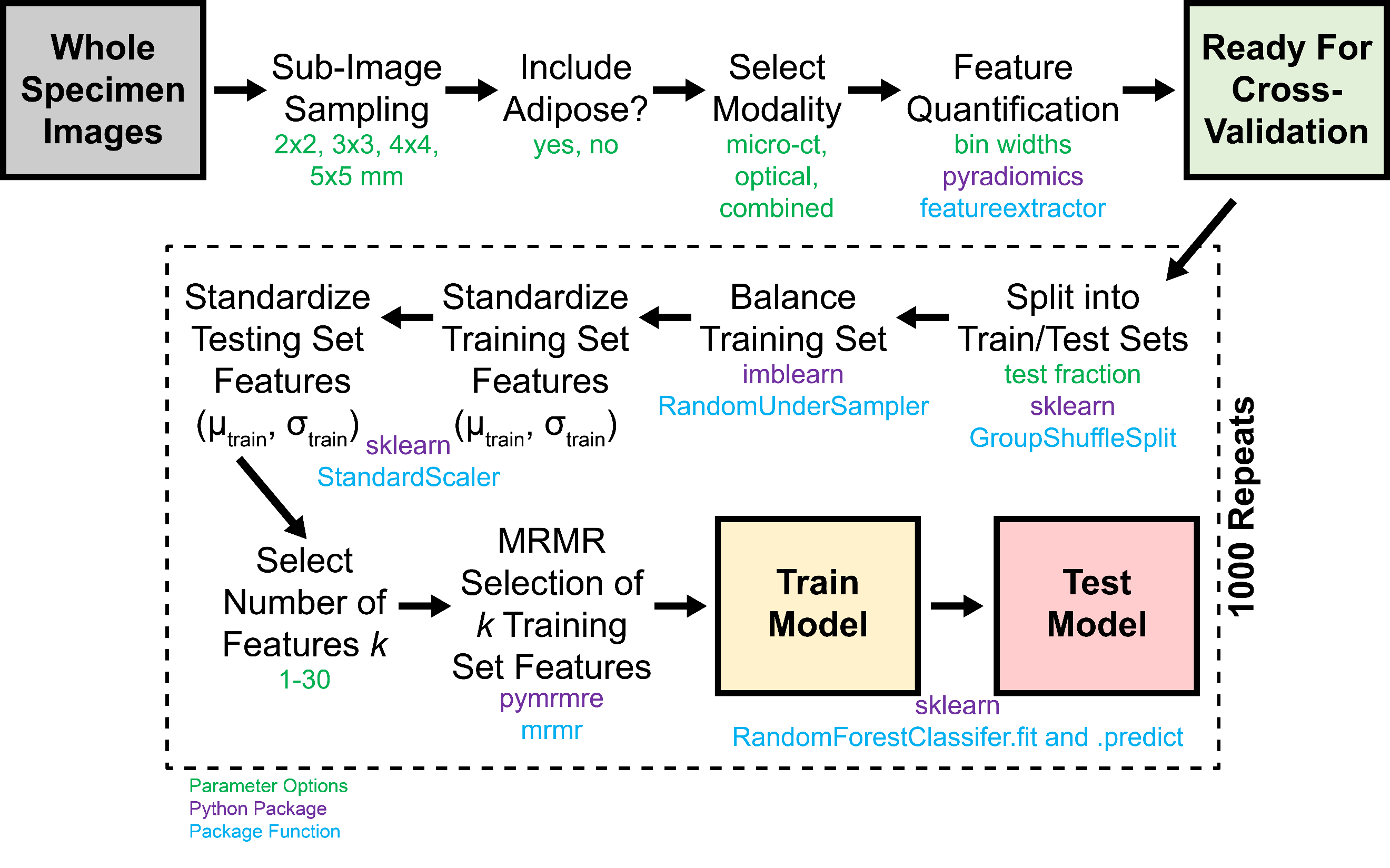


**Fig. S3** The supervised machine learning classification pipeline with patient-level Monte Carlo cross-validation. Key parameter options, Python packages, and Python functions are listed with green, purple, and blue text, respectively. MRMR = minimum redundancy, maximum relevance.


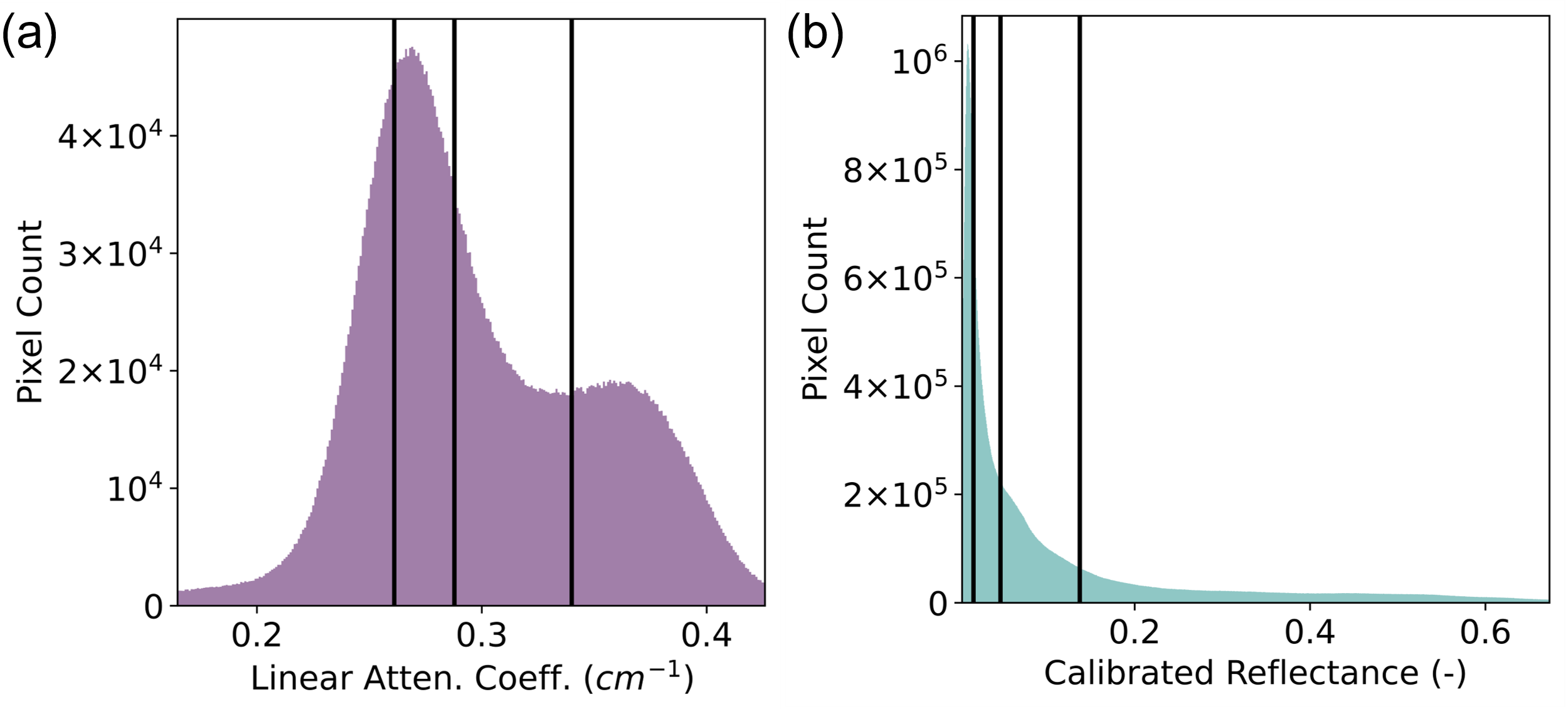


**Fig. S4** Histograms of all tissue pixels extracted from (a) the micro-CT slice images (one channel) and (b) the SFDI images (16 channels from every combination of four wavelengths and four spatial frequencies). Horizontal axis limits in each subplot represent the 1^st^ and 99^th^ percentile tissue pixel values, while the three vertical lines represent, from left to right, the 25^th^, 50^th^, and 75^th^ percentile values.

**Supplementary Material Tables**

**Table S1** Breast tumor sub-image sample totals by tissue subtype and sub-image size.

| **Tissue Subtype** | **2 x 2 mm** | **3 x 3 mm** | **4 x 4 mm** | **5 x 5 mm** |
| --- | --- | --- | --- | --- |
| **Healthy and abnormal benign** |  |  |  |  |
| Adipose tissue | 4,472 | 1,992 | 1,015 | 508 |
| Healthy connective tissue | 1,951 | 890 | 505 | 307 |
| Fibrocystic disease | 3,061 | 1,415 | 779 | 471 |
| **Invasive cancer** |  |  |  |  |
| Inv. ductal carcinoma low-grade | 514 | 237 | 118 | 64 |
| Inv. ductal carcinoma intermediate-grade | 898 | 419 | 231 | 132 |
| Inv. ductal carcinoma high-grade | 911 | 420 | 243 | 150 |
| Inv. lobular carcinoma | 1,095 | 507 | 282 | 170 |
| **Totals** | 12,902 | 5,880 | 3,173 | 1,802 |

**Table S2** Classification performance metrics derived from 5 x 5 mm sub-image samples, six optimal radiomic/optomic features per minimum redundancy, maximum relevance feature selection, and *n* = 1000 cross-validation splits (mean ± one standard deviation). AUC = area under the receiver operating characteristic curve.

|  | **Micro-CT Alone** | **Optical Alone** | **Combined** |
| --- | --- | --- | --- |
| **Adipose tissue included** |  |  |  |
| Accuracy | 0.82 ± 0.06 | 0.72 ± 0.09 | 0.84 ± 0.07 |
| Recall | 0.88 ± 0.08 | 0.79 ± 0.11 | 0.90 ± 0.08 |
| Precision | 0.87 ± 0.07 | 0.81 ± 0.11 | 0.88 ± 0.07 |
| AUC | 0.88 ± 0.07 | 0.78 ± 0.10 | 0.90 ± 0.06 |
| **Adipose tissue excluded** |  |  |  |
| Accuracy | 0.74 ± 0.09 | 0.70 ± 0.10 | 0.80 ± 0.08 |
| Recall | 0.82 ± 0.12 | 0.75 ± 0.14 | 0.85 ± 0.10 |
| Precision | 0.75 ± 0.14 | 0.74 ± 0.16 | 0.81 ± 0.13 |
| AUC | 0.78 ± 0.11 | 0.75 ± 0.12 | 0.85 ± 0.09 |

**Supplementary Material References**

1. Cuccia, D. J., Bevilacqua, F., Durkin, A. J. & Tromberg, B. J. Modulated imaging: quantitative analysis and tomography of turbid media in the spatial-frequency domain. *Opt Lett* **30**, 1354–1356 (2005).

2. Cuccia, D. J., Bevilacqua, F., Durkin, A. J., Ayers, F. R. & Tromberg, B. J. Quantitation and mapping of tissue optical properties using modulated imaging. *J Biomed Opt* **14**, 024012 (2009).

3. Kanick, S. C. *et al.* Sub-diffusive scattering parameter maps recovered using wide-field high-frequency structured light imaging. *Biomed Opt Express* **5**, 3376–3390 (2014).

4. McClatchy, D. M. *et al.* Wide-field quantitative imaging of tissue microstructure using sub-diffuse spatial frequency domain imaging. *Optica* **3**, 613–621 (2016).

5. Maloney, B. W. *et al.* Structured light imaging for breast-conserving surgery, part I: optical scatter and color analysis. *J Biomed Opt* **24**, 096002 (2019).

6. Krishnaswamy, V. *et al.* Structured light scatteroscopy. *J Biomed Opt* **19**, 070504 (2014).

7. Aerts, H. J. W. L. *et al.* Decoding tumour phenotype by noninvasive imaging using a quantitative radiomics approach. *Nature Communications* **5**, 4006 (2014).

8. Gillies, R. J., Kinahan, P. E. & Hricak, H. Radiomics: Images Are More than Pictures, They Are Data. *Radiology* **278**, 563–577 (2016).

9. van Griethuysen, J. J. M. *et al.* Computational Radiomics System to Decode the Radiographic Phenotype. *Cancer Res* **77**, e104–e107 (2017).

10. Pedregosa, F. *et al.* Scikit-learn: Machine Learning in Python. *Journal of Machine Learning Research* **12**, 2825–2830 (2011).

11. Xu, Q.-S. & Liang, Y.-Z. Monte Carlo cross validation. *Chemometrics and Intelligent Laboratory Systems* **56**, 1–11 (2001).

12. Lemaître, G., Nogueira, F. & Aridas, C. K. Imbalanced-learn: A Python Toolbox to Tackle the Curse of Imbalanced Datasets in Machine Learning. *Journal of Machine Learning Research* **18**, 1–5 (2017).

13. Radovic, M., Ghalwash, M., Filipovic, N. & Obradovic, Z. Minimum redundancy maximum relevance feature selection approach for temporal gene expression data. *BMC Bioinformatics* **18**, 9 (2017).

14. De Jay, N. *et al.* mRMRe: an R package for parallelized mRMR ensemble feature selection. *Bioinformatics* **29**, 2365–2368 (2013).

15. Parmar, C., Grossmann, P., Bussink, J., Lambin, P. & Aerts, H. J. W. L. Machine Learning methods for Quantitative Radiomic Biomarkers. *Scientific Reports* **5**, 13087 (2015).

16. Leijenaar, R. T. H. *et al.* The effect of SUV discretization in quantitative FDG-PET Radiomics: the need for standardized methodology in tumor texture analysis. *Sci Rep* **5**, 11075 (2015).

17. Tixier, F. *et al.* Intratumor heterogeneity characterized by textural features on baseline 18F-FDG PET images predicts response to concomitant radiochemotherapy in esophageal cancer. *J Nucl Med* **52**, 369–378 (2011).

18. McClatchy, D. M. 3rd, Zuurbier, R. A., Wells, W. A., Paulsen, K. D. & Pogue, B. W. Micro-computed tomography enables rapid surgical margin assessment during breast conserving surgery (BCS): correlation of whole BCS micro-CT readings to final histopathology. *Breast Cancer Res Treat* **172**, 587–595 (2018).
